# A Longstanding Pre-coccygeal Cyst With Recurrent Infection and Fistulisation: A Rare Case of a Presacral Lesion in a Young Woman

**DOI:** 10.7759/cureus.86776

**Published:** 2025-06-26

**Authors:** Swapnil M Saraiya, Rudransh Guleria

**Affiliations:** 1 General Surgery, University Hospital of North Tees and Hartlepool NHS Trust, Stockton on Tees, GBR

**Keywords:** affect of hormonal changes on cyst, congenital epidermoid cyst, duplication cysts, presacral (retrorectal) cysts, surgical approaches to presacral lesions

## Abstract

Presacral cystic lesions are rare and diagnostically challenging due to their nonspecific symptoms and deep anatomical location. We report a case of a young woman with a pre-coccygeal cyst, first identified following an emergency Caesarean section in 2020. Over the next five years, the lesion increased in size, likely exacerbated by hormonal changes during successive pregnancies, resulting in chronic pelvic pain, neurological symptoms, and cutaneous fistulisation.

Imaging via MRI and CT demonstrated a complex, encapsulated lesion with peripheral enhancement and restricted diffusion. Multidisciplinary evaluation suggested a congenital epidermoid or duplication cyst. Given the absence of malignancy and surgical complexity, a conservative management strategy was adopted.

This case underscores the importance of including longstanding presacral cysts in differential diagnoses and highlights the value of multidisciplinary input and long-term surveillance in guiding management.

## Introduction

Presacral (retrorectal) cysts are rare lesions located in the potential space between the rectum and sacrum. They encompass a heterogeneous group, including congenital, neurogenic, osseous, and inflammatory origins [1]. Congenital variants, such as epidermoid and duplication cysts, are typically benign and arise from embryological remnants [2]. Duplication cysts are defined by a characteristic epithelial lining and muscular wall structure. They occur more frequently in women and often remain asymptomatic unless complicated by infection or progressive enlargement [3].

Due to their deep pelvic location, presacral lesions often present late, with nonspecific symptoms like back pain, pelvic pressure, or neurological disturbances [4]. Differentiating congenital cysts from chronic abscesses or malignancies remains challenging, especially when imaging findings overlap. Hormonal changes during pregnancy, specifically elevations in oestrogen, progesterone, and growth hormone, can stimulate epithelial proliferation and contribute to cyst growth.

MRI remains the gold standard imaging modality, allowing detailed characterisation of lesion architecture, wall thickness, and diffusion properties [5]. However, definitive diagnosis frequently requires histopathological correlation, as imaging cannot reliably distinguish benign cysts from chronic abscesses or neoplasms [6]. While surgical excision is definitive, conservative management may be justified in selected cases, depending on symptomatology, anatomical complexity, and risk stratification [7].

We present a rare case of a longstanding pre-coccygeal cyst complicated by recurrent infection and fistulisation, with symptom progression following multiple pregnancies. This case highlights the importance of multidisciplinary evaluation and cautious, individualised management.

## Case presentation

**Figure 1:**
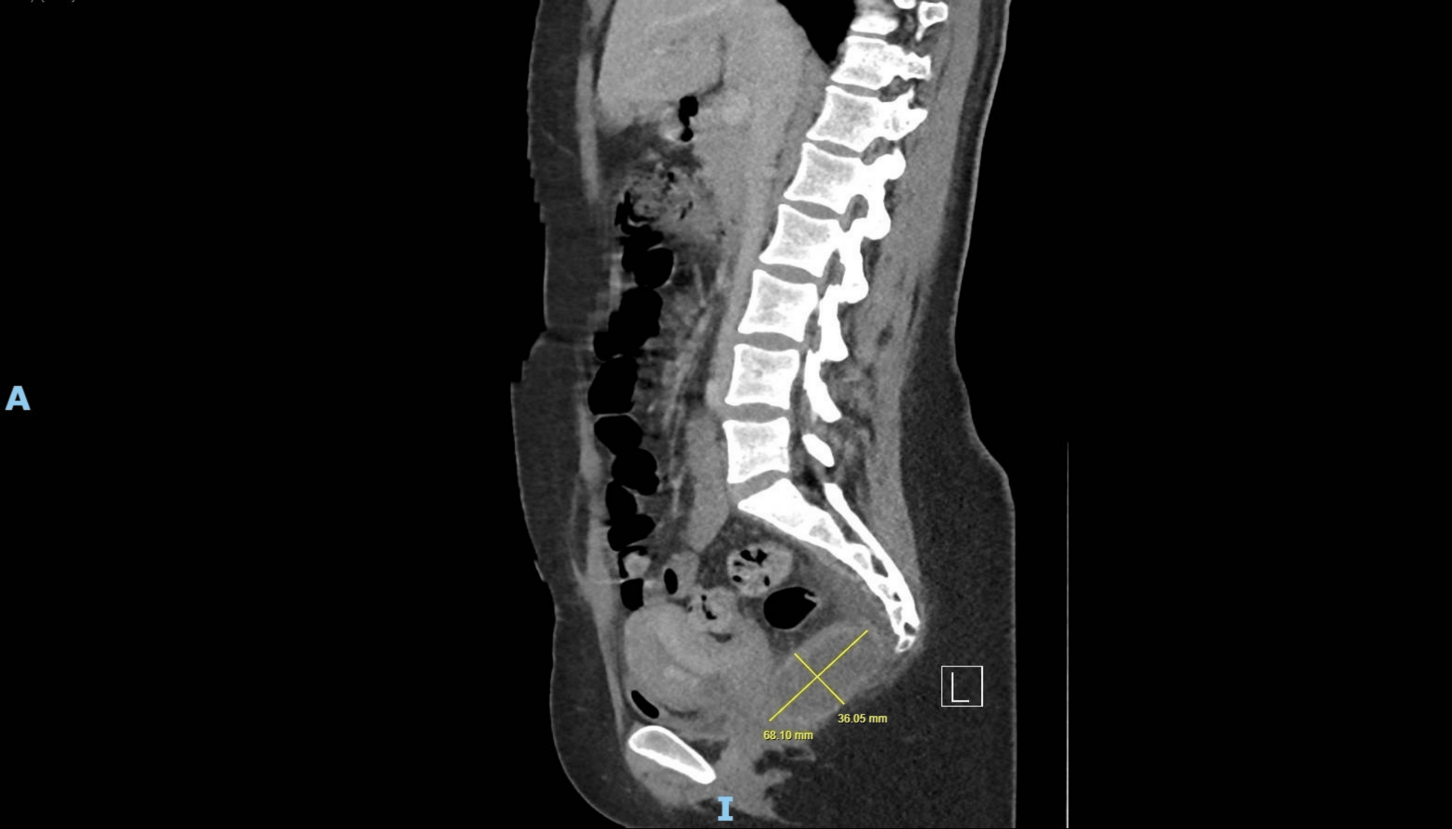
Sagittal CT pelvis (2020) showing pre-coccygeal lesion with peripheral enhancement and proximity to the rectum and coccyx

**Figure 2:**
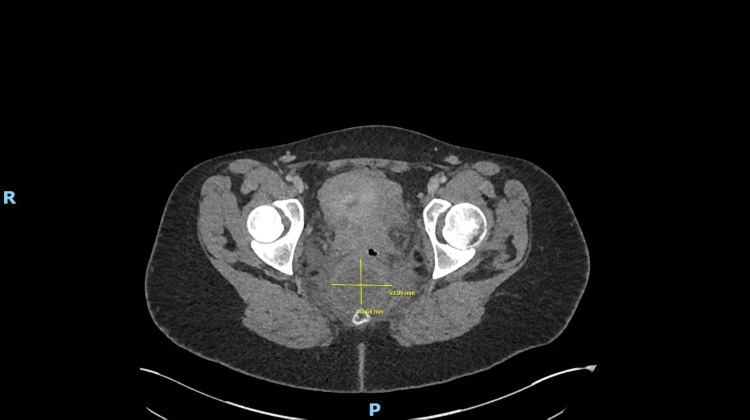
Axial CT pelvis (2020) showing lesion at initial diagnosis with adjacent structures

**Figure 3:**
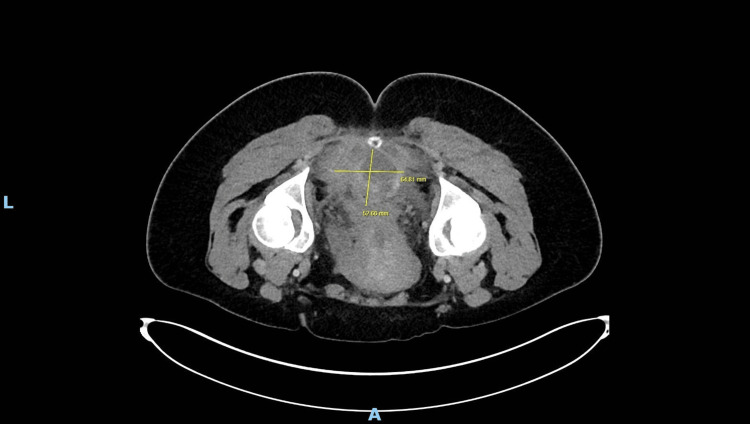
Axial CT pelvis post-aspiration (2020) illustrating residual cystic lesion after aspiration procedure

A young woman presented in 2020 with fever, lower back pain, and elevated inflammatory markers (CRP 215 mg/L) shortly after an emergency lower segment Caesarean section. CT imaging of the abdomen and pelvis revealed a 7 × 5 × 5 cm pre-coccygeal cystic lesion with thickened walls, internal septations, and peripheral enhancement, closely abutting the rectum and coccyx (Figures 1, 2). CT-guided aspiration yielded thick purulent material, indicating superimposed infection (Figure 3). The procedure was limited due to patient discomfort and sciatic nerve irritation. The patient improved with intravenous antibiotics and was discharged.

Over the next five years, she reported intermittent pelvic pain, intermittent paraesthesia and numbness in the right lower limb, and discomfort when sitting. A mucoid discharge occasionally appeared from a sinus in the natal cleft. Her medical history included a spinal abscess in 2020 which was dealt with long-course antibiotics. She had three pregnancies between 2020 and 2025, potentially contributing to lesion progression.

**Figure 4:**
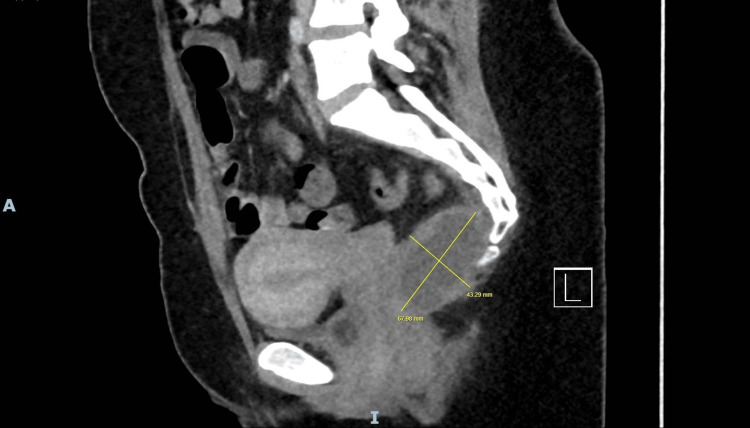
Sagittal CT pelvis (2025) demonstrating progressive enlargement of presacral cystic lesion extending into ischiorectal fossa

**Figure 5:**
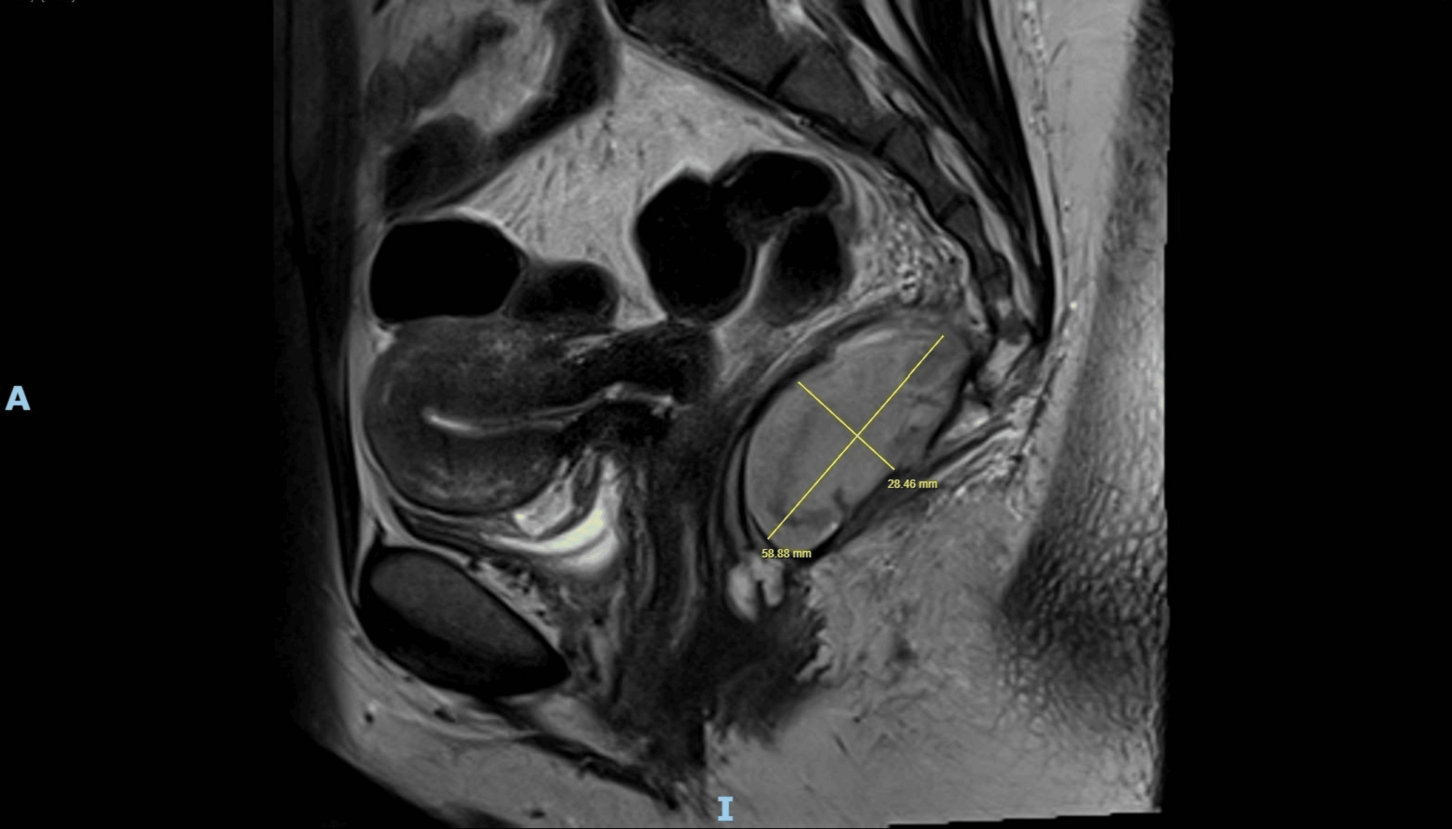
Sagittal MRI pelvis (2025) illustrating lobulated, T2-hyperintense presacral cystic lesion with peripheral enhancement

**Figure 6:**
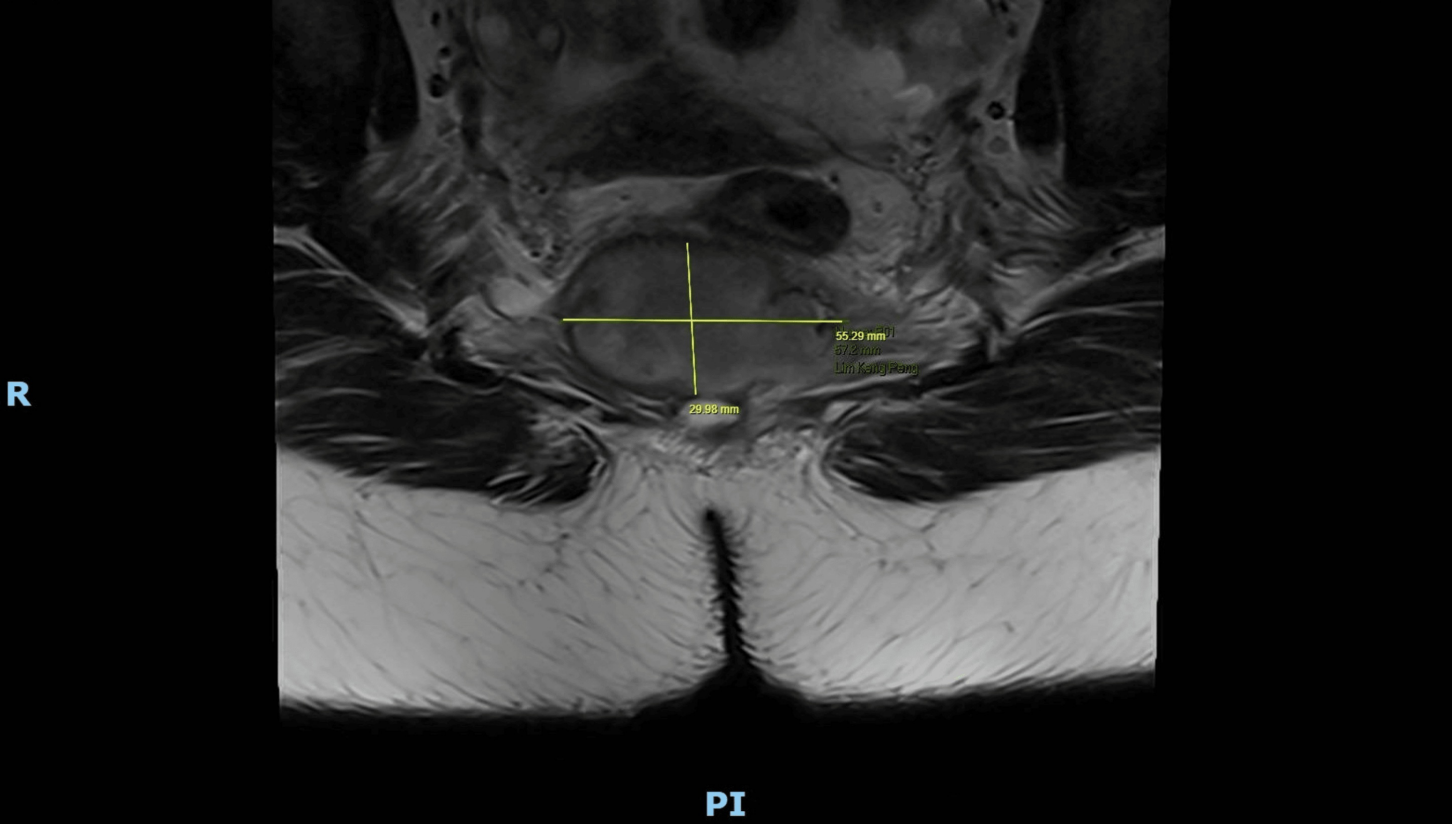
Axial MRI pelvis (2025) highlighting lobulated lesion with restricted diffusion and peripheral enhancement extending into surrounding soft tissue

In 2025, she re-presented with exacerbated pelvic pain, urinary urgency, and constipation. CT thorax-abdomen-pelvis confirmed progression to a 65 × 38 mm presacral lesion (Figure 4). MRI pelvis revealed a lobulated, encapsulated 79 × 41 × 59 mm T2-hyperintense lesion with mildly restricted diffusion pattern and peripheral enhancement (Figures 5, 6). Extension into the left ischiorectal fossa through the levator ani was noted without bony erosion or lymphadenopathy.

**Figure 7:**
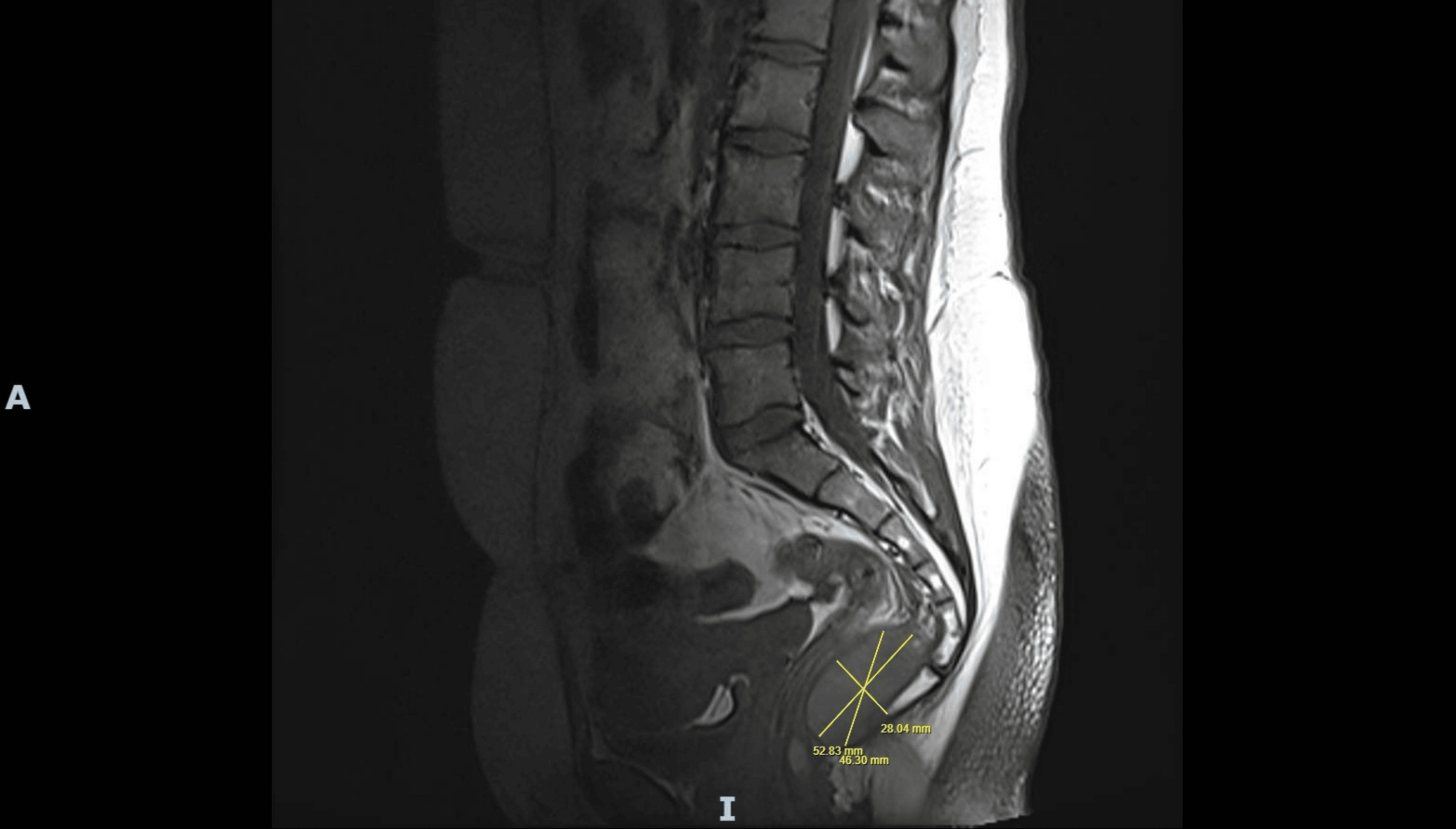
Sagittal MRI of pelvis (2025) demonstrating presacral cystic lesion with thickened walls and internal septations

Regional colorectal and sarcoma multidisciplinary teams reviewed the case. Differential diagnoses included infected epidermoid cyst, duplication cyst, or chronic abscess. Examination under anaesthesia identified a mature sinus tract; aspiration yielded 3 mL of sterile fluid. Cytology was negative for malignancy, tuberculosis, and fungal infection (Figure 7).

Given the chronic, slow progressive course, absence of malignancy, and potential surgical complications, conservative management was chosen. The patient commenced treatment with antibiotics, analgesic under regular pain clinic review, with structured outpatient follow-up and multidisciplinary team discussion.

## Discussion

Presacral cysts are rare, occurring in approximately one in 40,000 hospital admissions [1]. Congenital types such as epidermoid and enteric duplication cysts are thought to arise from incomplete embryological regression and are typically lined with squamous or columnar epithelium [2,3].

In this case, the cyst remained quiescent until detection postpartum which was already known to us before preganancy. Subsequent pregnancies likely promoted cystic growth, influenced by increased levels of oestrogen and growth hormone, both of which are known to stimulate epithelial proliferation.

MRI findings were consistent with an infected congenital cyst, showing restricted diffusion attributable to keratinaceous or mucinous content, and peripheral enhancement reflective of inflammation [5]. However, given imaging limitations in excluding malignancy, histopathological confirmation was essential [6].

While complete surgical excision is standard for symptomatic lesions - particularly when malignancy is suspected or neurological compromise exists - conservative management is a viable alternative in selected patients [4,7]. In our patient, the risk of pelvic nerve damage, anal sphincter injury, and technical difficulties during resection were significant. Given the absence of malignant features, a non-operative approach prioritising symptom control and surveillance was chosen.

Retrorectal cysts are histologically classified based on embryological origin. These include epidermoid, dermoid, enteric duplication cysts, cystic hamartomas, and teratomas [8]. Two theories explain their embryogenesis: the Veeneklass theory attributes origin to failed notochord separation, accounting for heterotopic epithelium; the Lewis-Thyng theory suggests cyst formation arises from fetal diverticula during early gestation [9].

Rectal duplication cysts are the rarest and characterised by squamous epithelial lining, smooth muscle components, mucin-secreting transitional epithelium, and occasional lymphoid aggregates or apocrine elements [10].

Surgical approaches to presacral lesions depend on size, location, and complexity, and range from transanal and transcoccygeal to abdominoperineal resections [9]. Minimally invasive techniques, such as laparoscopic total mesorectal excision or transanal endoscopic microsurgery, may be suitable in carefully selected cases [11]. The mention of transanal minimally invasive surgery (TAMIS) reflects a potential surgical option for similar lesions, though it was not performed here. Future management may reconsider surgical intervention if symptoms worsen.

## Conclusions

Presacral cysts are uncommon congenital lesions that often remain asymptomatic for long periods, typically presenting in adulthood with nonspecific symptoms, especially among women. Hormonal changes during pregnancy may lead to cyst enlargement and symptom exacerbation. Magnetic resonance imaging (MRI) plays a critical role in the characterization and evaluation of these cysts, though definitive diagnosis often necessitates histological examination to exclude malignancy. Multidisciplinary management and structured follow-up are essential to achieving optimal patient outcomes and personalized care.

Rectal duplication cysts (RDCs) are rare congenital anomalies located within or adjacent to the rectum, characterized by clinical presentations such as rectal pain, bleeding, tenesmus, or alterations in bowel habits. Due to their rarity and typical submucosal or extramural positions, RDCs can be misdiagnosed as other rectal or presacral pathologies. Diagnostic imaging techniques, particularly MRI and endoscopic ultrasound (EUS), facilitate preoperative assessment; however, definitive diagnosis generally requires surgical excision and subsequent histopathological analysis.

Complete surgical resection remains the treatment of choice to establish a definitive diagnosis and prevent complications, including infection, fistula formation, or malignant transformation. TAMIS represents a safe, effective, and minimally invasive surgical modality, increasingly utilized for both diagnostic and therapeutic management of these lesions.

## References

[1] Hobson KG, Ghaemmaghami V, Roe JP, Goodnight JE, Khatri VP (2005). Tumors of the retrorectal space. Dis Colon Rectum.

[2] Dozois EJ, Marcos MD (2011). Presacral tumors. The ASCRS Textbook of Colon and Rectal Surgery.

[3] Hassan I, Wietfeldt ED (2009). Presacral tumors: diagnosis and management. Clin Colon Rectal Surg.

[4] Lev-Chelouche D, Gutman M, Goldman G (2003). Presacral tumors: a practical classification and treatment of a unique and heterogeneous group of diseases. Surgery.

[5] Yang DM, Jung DH, Kim H, Kang JH, Kim SH, Kim JH, Hwang HY (2004). Retroperitoneal cystic masses: CT, clinical, and pathologic findings and literature review. Radiographics.

[6] Kang HC, Menias CO, Gaballah MH (2018). Beyond the bowel: mesenteric and omental cystic masses of gastrointestinal origin. Radiographics.

[7] Baek SK, Hwang GS, Vinci A, Jafari MD, Jafari F, Moghadamyeghaneh Z, Pigazzi A (2016). Retrorectal tumors: a comprehensive literature review. World J Surg.

[8] Mathis KL, Dozois EJ, Grewal MS, Metzger P, Larson DW, Devine RM (2010). Malignant risk and surgical outcomes of presacral tailgut cysts. Br J Surg.

[9] Au E, Anderson O, Morgan B, Alarcon L, George ML (2009). Tailgut cysts: report of two cases. Int J Colorectal Dis.

[10] Dallal WA, Narayanasamy S, Ramasamy S, Mohamedahmed AY, Husain N (2024). Rectal duplication cyst presenting with change in bowel habits and rectal bleeding: a case report and literature review. Surg Case Rep.

[11] Duek SD, Gilshtein H, Khoury W (2014). Transanal endoscopic microsurgery: also for the treatment of retrorectal tumors. Minim Invasive Ther Allied Technol.

